# The Impact of Water and Other Fluids on Pediatric Nephrolithiasis

**DOI:** 10.3390/nu14194161

**Published:** 2022-10-07

**Authors:** Carmen Iulia Ciongradi, Florin Filip, Ioan Sârbu, Codruța Olimpiada Iliescu Halițchi, Valentin Munteanu, Iuliana-Laura Candussi

**Affiliations:** 1 2nd Department of Surgery—Pediatric Surgery and Orthopedics, “Grigore T. Popa” University of Medicine and Pharmacy, 700115 Iași, Romania; 2 Pediatric Surgery and Orthopedics Department, County Hospital, “Ștefan cel Mare” University, 720229 Suceava, Romania; 3 Department of Mother and Child Medicine-Pediatrics, “Grigore T. Popa” University of Medicine and Pharmacy, 700115 Iași, Romania; 4 Department of Biomedical Sciences, “Grigore T. Popa” University of Medicine and Pharmacy, 700115 Iași, Romania; 5 Clinical Surgery Department, Faculty of Medicine and Pharmacy, “Dunărea de Jos” University, 800008 Galați, Romania

**Keywords:** nephrolithiasis, water, prevention, hydration, pediatric, pediatric nephrology, pediatric surgery

## Abstract

Pediatric nephrolithiasis cases have been on the rise in the past several years, resulting in increased healthcare costs and other burdens on the juveniles with this ailment. Recent research has shown that present trends in pediatric nephrolithiasis have changed as a result of fluid intake, including water consumption, nutrition, obesity prevalence, lifestyle, and imaging procedures. A specific cause, meanwhile, is still elusive. Trends in pediatric nephrolithiasis need to be thoroughly researched. Furthermore, variables specific to pediatric nephrolithiasis that could cause greater difficulties in an affected child elevate the level of worry with cumulative prevalence. Doctors should rigorously assess patients who present with kidney stones when they have dynamics such as varied clinical presentation, high recurrence of kidney stones linked to metabolic and urinary tract problems, and the potential existence of rare genetic kidney stone illnesses. This review aims to identify adaptive risk factors and anomalies that call for specialized treatment and prescription. More specifically, the major goals of medical and surgical treatments are to eliminate kidney stone risk and stop relapse while concurrently lowering interventional barriers. A dedicated nephrolithiasis clinic run by a pediatric nephrologist, nutritionist, urologist, and clinical nurse may sometimes be beneficial for patients in serious danger. Such a clinic offers significant chances to learn more about pediatric nephrolithiasis, which has been linked to water consumption and hence fosters urgently required study in this area.

## 1. Introduction

Urolithiasis (UL) is a multifactorial disease that affects men and women of all ages. It is characterized by the accumulation of calculi in the urinary tract [1]. Calculi typically consist of accumulated uric acid or calcium oxalate but may also be formed from several different substrates and other minerals and acids such as struvite [2,3]. While it is usually diagnosed in adulthood, about 10% of all cases involve pediatric patients [4]. No treatment for removing calculi from pediatric patients reduces or modifies this morbidity. Recurrence is observed in up to 50% of cases [5]. UL may be caused by infections, but metabolic factors are increasingly responsible for development and recurrence [6,7]. UL is directly linked to morphological alterations and surgical interventions occurring due to urinary tract blockades as well as to their clinical symptoms [8]. A particular challenge with the clinical management of UL in children is the lack of appropriate diagnostic tools to assess the presence of calculi and dietary status [9].

The incidence, clinical features, and structure of juvenile UL differ according to geographic location. Such variance is thought to be due to socioeconomic, climatic, and dietary factors, including drinking water and the regular consumption of specific liquids like cranberry juice, tea, blackcurrant juice, or plum juice, among others [8]. In approximately 70–80% of cases, the calculi resolve spontaneously or are excreted, with the remaining cases requiring surgical removal [10]. Even if the calculi are spontaneously excreted, recurrence is a matter of concern and, hence, treatment strategies focus on the prevention of repeated stone formation [11]. Dietary factors, particularly those affecting fluid balance and osmolarity, are of relevance in the prevention of stone formation [12]. Therefore, the aim of the present review is to summarize the available studies on the role of dietary fluids in pediatric UL as a basis for future clinical guidelines. The review discusses the requirements for assessing hydration status in the pediatric population and the methods available to assess hypertonic dehydration status. The consequences of various fluids on the hydration status of children with UL caused by suboptimal fluid intake are summarized.

## 2. Water and Diet

In recent times, the clinical guidelines of both the American College of Physicians (ACP) and the American Urological Association (AUA) have recommended sufficient water intake for the prevention of recurrent kidney stones [13]. However, the correlation between fluid ingestion and UL risk is not entirely clear yet. Previous studies have suggested that adequate fluid (mostly water) consumption is an effective strategy to prevent the occurrence of kidney stones [13]. Furthermore, three meta-analyses concluded that water ingestion is associated with diminished UL risk and diminished long-term risk of recurrence [13]. Nevertheless, some studies report conflicting results [14]. This meta-analysis objectively defined the quantitative relationship between high water intake and uric acid nephropathy (UAN) risk [14]. Neither the clinical guidelines nor the meta-analysis addressed the relationship between high water intake and prevention of UAN.

It is evident that diet is a risk factor for UL and that adaptation of dietary patterns could be an effective treatment option [14]. This is true not only for adult patients with UL but also for younger patients. Yilmaz and Ünal recently showed that consumption of milk and dairy products could be associated with increased UL risk in infants [15]. In line with this, Bozkurt et al. discovered that the breastfeeding of infants with renal stones may decrease the stone size and halt the progression of the disease [16]. Sarica et al. also observed a beneficial effect of breastfeeding for the clinical course of children with UL [17].

Low fluid consumption and excessive sodium intake have been determined to be the most relevant risk factors for developing UL and calcium kidney stones in adults. The endorsements for dairy products and calcium have changed from restraining these fluids to boosting their consumption at the recommended dietary allowance (RDA) for the public after three studies demonstrated increased risk of stones with low calcium intake in adults [18]. The hypothesis is that reduced calcium in the diet allows more absorption of oxalate, an outcome that may have even greater effects on calcium oxalate stone formation than intake of calcium alone.

Although the intricate details of UL are unclear, hyper-saturation of urine is required for the formation of urinary stones. The process of crystallization begins when a certain solute supersaturates urine. However, if the solution is unsaturated, no crystals can form. The supersaturation depends on several variables, including the ineffectiveness of crystallization inhibitors (such as magnesium, citrate, nephrocalcin, pyrophosphate, and glycosaminoglycans), persistently acidic or alkaline urine pH, and hypersecretion states of uric acid, oxalate, calcium, phosphorus, and cystine [19].

Furthermore, the equilibrium between water output and input defines hydration status. Miller and Stapleton [20] found that the urine volume of children with UL was significantly lower than that of controls without UL. In line with this, Penido et al. [21] observed a very low urine output in pediatric UL patients.

It is assumed that the correlation between low fluid intake and the development of UL is linked to the osmolality of the tissues and, hence, the concentration of certain minerals and acids in the blood. Velasquez-Forero et al. [22] assessed risk factors for pediatric UL in a group of 162 children with UL. The authors identified low blood levels of citrate and magnesium and high levels of calcium in the blood as the most important risk factors for the development of urinary stones in children [22]. Compared to the controls, children with UL also had significantly higher levels of uric acid and oxalates and lower levels of albumin and phosphorus. Kovacevic et al. [23] also identified hypocitraturia and hypercalcemia as the most prevalent risk factors associated with UL in children. Turudic et al. [24] assessed the excretion of calcium, oxalate, and citrate by children with and without UL and found a link between low citrate and high calcium urinary excretion and the development of UL. Turudic et al. also pointed out that the ratios of calcium and citrate as well as oxalate/(citrate X glycosaminoglycans) may be useful as diagnostic criteria to detect UL in the pediatric population [25]. MacDougall et al. [26] conducted a case-control study and also detected lower levels of citrate and higher levels of calcium in the urine of children with urolithiasis. Citrate levels in the urine are influenced by the acid-base status of the body, which in turn is influenced by the dietary consumption of acidic and alkaline foods [27,28].

For the patient with calcium oxalate stones and hypercalciuria, treatment will be nonspecific or guided by the outcomes of diet assessment and a 24-h urine sample. The primary recommendation is to increase fluid intake, which increases the urine output. The recommended daily urine outputs, by age, are: infants, 750 mL or more; young children younger than 5 years of age, 1000 mL or more; children between the ages 5 and 10 years, 1500 mL or more; and children older than age 10 years, 2000 mL or more. The recommended daily intake must be about 50% greater than the targeted output to account for other losses. Specific advice should be for the patient and their family, in regard to suggested servings, to incorporate 8 to 20 oz. at sleep time to evade low urine output overnight.

Moreover, the danger of stones in adults can be reduced up to 50%, the so-called stone clinic effect with an increase in urine output alone. The second recommendation for children with hypercalciuria is to cut back sodium intake to a “no added salt” diet; in industrialized countries, 75% of salt in the diet comes from salt added to processed food. Other recommendations include adequate potassium intake and a moderate amount of animal protein consumption. For the patient with hyperoxaluria without a primary cause, such as enteric hyperoxaluria, the primary step is to ensure an adequate calcium intake in accordance with the recommended daily allowance (RDA). High-fat diets should be avoided because lipids bind enteric calcium and promote oxalate absorption. Moderate restriction of high-oxalate foods is generally suggested. Finally, avoidance of excess ascorbic acid, which is metabolized to oxalate within the body, is recommended. For children for whom dietary therapy is ineffective in regulating UL, laboratory analysis of a 24-h urine sample is needed to guide pharmacologic therapy. A thiazide diuretic may be required for a child with hypercalciuria and a lack of response to a sodium-restricted diet. The standard recommendation is hydrochlorothiazide 1 to 2 mg/kg per day. To avoid hypokalemia, add amiloride, which is potassium-sparing and has an extra hypocalciuric effect. For the child who has low citrate excretion, potassium citrate at a dose of two to three mEq/kg per day for infants or younger children or 30 to 80 mEq/day for older children and adolescents is often added in two divided doses, with the second dose at bedtime. This regimen may be useful in preventing hypokalemia in the child who is also receiving a thiazide diuretic. Potassium citrate is also indicated to alkalinize the urine in children who have RTA and orthophosphate stones and for the rare patient who has low urine pH and acid stones [29].

One study specifically compared oligo-mineral water with bicarbonate-alkaline high-calcium mineral water, and concluded that water with a reduced concentration of calcium should be consumed, as calcium-rich water increases the chance of nephrolithiasis. The current literature supports this finding, as in the UK it has been recommended that drinking water is suitable owing to its low calcium levels [30,31,32].

## 3. Prevention of Recurrence

Prevention of nephrolithiasis recurrence in children entails identification of the potential risk factors of stone formation and their modification. Previous reports indicated that children with known underlying metabolic disorders have a 50% risk of recurrence of nephrolithiasis, compared to 10% in patients without these risk factors. A suitable fluid intake of 1.5 L/m^2^ per day and a restricted dietary intake should be used to prevent supersaturation of urinary rudiments like uric acid, calcium, and oxalate. The fluid intake could be enough to provide a urine volume of more than 750 mL/d in infants and more than 1000 mL/d for children less than five years old, 1500 mL/d for children 5 to 10 years, and 2000 mL/d for children more than 10 years old. Children need appropriate dietary calcium for bone formation; calcium restriction is, therefore, not recommended. Excessive protein intake can lead to hypercalciuria and hypocitraturia; hence, protein intake should follow current recommendations. Furthermore, avoidance of medication that may cause nephrolithiasis should be considered (Table 1) [33].

## 4. Soda Intake

A correlation between soda-based beverages and UL is questionable [38]. The discontinuation of such drinks was demonstrated to be significantly protective against UL, particularly in the case of soda drinks containing phosphoric acid, while some studies in adult populations found no association [38]. For children, it might be appropriate to allow soda drinks only on special occasions, although tight dietary restrictions are not recommended. First, such restrictions will make it more difficult for people to take their medications, and second, they will produce nutritional deficiencies that are more serious than the UL itself (reduced bone mineral density, height and weight loss, multiple vitamin deficiency, etc.). Therefore, the diet should be suitable for the child or adolescent and changed in accordance with individual nutritional requirements as well as the RDA for calories, proteins, and calcium [8].

## 5. Milk, Fruit Juices, and Artificial Drinks Containing Carbohydrates

Children between the ages of 4 and 8 should consume 1.2 g of sodium daily, while those between the ages of 9 and 18 should consume 1.5 g. The comparable upper limits are 1.9 g and 2.3 g, and anything over those amounts may also be associated with health risk [18]. Typically, dairy products, vegetables, and fruit contain potassium. There are different recommendations for the consumption of these foods according to age: 3.8 g for children aged 4 to 8, and 4.5 g for those aged 9 to 18. This frequently corresponds to three units per day. Urine Na/K ratio testing, which should be under 2.5, can be used to monitor proper consumption of those components. Excessive consumption of foods high in sodium is associated with higher natriuresis, which may promote hypercalciuria, a condition that predisposes one to developing stones [18]. In case of an insufficient urine finding, all patients with hypercalciuria should have their Na/K ratio examined, and natriuria should be taken into consideration as a very important dietary factor to be adjusted. Reduced intake of animal-derived protein (such as red meat) is another potential dietary change. The increased acidity that results from protein metabolism should be balanced by bone-released bicarbonate. Hypercalciuria and reduced bone mineral density can develop when bone resorption is severe. Additionally, the consumption of other sugars (sucrose, fructose), of vitamins (vitamin C), and of minerals like magnesium and phytate may prevent the formation of calculi [39].

Popkin et al. [34] suggested that the replacement of water with sugar-sweetened beverages, juice, and milk is related to a reduced energy intake. The literature concerning the effect of water intake on energy intake in children is extremely limited, but a German school intervention study with water recommended that the water consequences on general energy intake of youngsters are comparable to those in adults [35].

The results of a study by Piero et al. confirmed that a standard diet without juice supplementation is equally effective in lowering the risk of recurrent stones in patients with calcium oxalate nephrolithiasis as a standard diet with standard calcium content but reduced amounts of salt and animal proteins. The results were most likely skewed by the decreased compliance of the patients with the advised juice supplementation during the two-year follow-up period. In fact, a conventional diet with juice supplementation was believed to be more protective against stone recurrence than a normal diet without supplementation when the follow-up was closed at one year after randomization, when patient adherence was still at 68%. The treatment effect was significant even after statistical adjustments for the three predefined potential risk factors of age, sex, and citraturia stratum. The baseline characteristics of the study patients, including urine volume, urine pH, and urinary solutes concentration at baseline were similar among the treatment groups, and each of the patients was recommended the identical standard diet. Thus, the study findings were unlikely to have been confounded by an unbalanced distribution of risk factors for stone recurrence within the two study groups [36].

In a recent review, Armstrong has reported that urolithiasis is that the only disorder that has consistently been related to chronic low daily water intake, whereas evidence suggests that in conditions like obesity and type 2 diabetes, increased water intake may reduce energy intake in some individuals [37].

In total, six types of water (oligo-mineral water and bicarbonate-alkaline high-calcium drinking water [40], H_2_O [41] de-ionized water [41], Fiuggi mineral water, and tap water [42]) and other types of beverages (blackcurrant juice, fruit juice, plum juice, fruit crush [43], lemonade [44], fruit crush, fruit crush, caffeine-free diet Coke, Fresca, and Gatorade [42]) were examined. Di Silverio et al. [42] found that there was a 6% higher recurrence of nephrolithiasis in participants who consumed water with high calcium content than in people who consumed Fiuggi water. Coen et al. [40] saw a large increase in urinary calcium excretion following the consumption of bicarbonate-alkaline-calcium–rich drinking water, together with a better relative supersaturation for CaOx stones. De La Guéronnière et al. [45] found that increasing fluid intake exerts a protective effect by reducing the crystallization index in the urine, a finding that was also supported by Di Silverio et al. [42]. Kessler et al. [43] found that blackcurrant juice alkalinized urine, which could prevent acid stones, and increased acid excretion, whereas fruit juice acidified urine and decreased acid excretion. There were no apparent changes observed in urinary parameters following the ingestion of plum juice. The results of a case-control study analyzing fruit juices was in agreement with the finding that they acidify urine. However, fruit juice was also found to significantly increase calcium and oxalate levels. After consuming grapefruit, orange, and apple juices, the relative supersaturation risk (RSR) for CaOx stones decreased, and a rise in acid excretion was observed with all three juices [44]. In their investigation, Odvina et al. [41] discovered a similar outcome for fruit juice.

Looking at age and sex, a study of the National Health and Nutrition Examination survey indicated that only 15–25% of children fulfil their overall water intake targets [41]. Furthermore, water consumption alone accounts for only a small fraction of the total fluid intake in these children, with milk, fruit juices, sports drinks, and sodas being consumed in larger quantities [41]. Less well-defined, but likely equally important, is the impact of dietary choices on urinary stone risk. Daily sodium consumption, a known contributor to hypercalciuria in children, often exceeds the recommended daily allowance. Furthermore, sodium intake may drive thirst response, and higher daily sodium intake has been associated with an increased intake of sugar-sweetened beverages in children [44].

Children should drink enough to provide 35 mL of urine per kg per day, while adults are advised to stay well-hydrated to produce 2.5 L of urine der day [46]. The hyper-saturation of calcium oxalate, phosphate, and acid is reduced when urine flow increases [47]. Children should have access to school team data so they can take water bottles and take more frequent restroom breaks. The type of fluid will determine how likely it is to prevent nephrolithiasis. Drinking sugary beverages, such as soda or beverages sweetened with high fructose corn syrup, has been linked to an increased risk of developing kidney stones [48]. In certain trials [49], but not in others [41,50], drinking lemonade has been shown to lessen recurrence in children with hypocitraturic nephrolithiasis. The cation must be present. When food or drink is almost entirely acidic, citrate is neutralized and does not raise urinary citrate levels. Urinary citrate can rise when consumed with meals and drinks high in potassium citrate, such as orange juice and some powdered lemonade mixes. Additionally, calorie consumption must be considered with these beverages.

For young children, sodium consumption should be limited to 2–3 mEq/kg/day, and for adults or adolescents to 2.4 g [51]. Furthermore, one level teaspoon of salt contains around 2300 mg of sodium. A boost in salt consumption will directly intensify the competition between calcium and sodium for passive reabsorption along the nephron. A rise in sodium consumption will immediately worsen calciuria because sodium and calcium compete for passive reabsorption along the nephron. If the child is in a normal state of hydration and no diuretics are being used, the amount of salt in the urine mirrors the sodium in the meal. Consumption of calcium cannot be restricted. In fact, high dietary calcium intake has been linked to a lower risk of stones in adults, but low-calcium diets have been linked to an increased risk of nephrolithiasis [52]. This is probably due to the ability of dietary calcium to reduce oxalate absorption through the digestive tract.

Adults who are obese are more likely to develop stones both before and after surgery. In a recent study with obese teenagers, it was discovered that oxalate excretion increased significantly following Roux-en-Y gastric bypass surgery compared to a group that underwent sleeve gastrectomy [53]. When comparing a traditional calcium, low-animal-protein, low-salt diet to a conventional low-calcium diet [54], Class I, Borghi et al. discovered a reduction in the recurrence of calcium oxalate stones. It is believed that lowering the consumption of purines provides the advantage suggested by low-animal-protein recommendations. Urinary acidity is raised by purines. Protein-rich diets may also raise the fixed acid load, which could lead to an increase in bone reabsorption and hypercalciuria. Additionally, consuming large amounts of animal protein may cause hyperoxaluria by increasing intestinal oxalate absorption and stimulating endogenous oxalate synthesis [47]. Children should not be subjected to protein restrictions while they are growing, but too much protein consumption should also be avoided.

The main observations from these studies are as follows:

- Certain fluids such as grapefruit, apple, and orange juices reduce urine calcium oxalate saturation, with a subsequent reduction in stone formation.
- Higher fluid intake is related to an increased urine output and reduced stone formation.

Fluids low in calcium seem to scale back the danger of renal calculus disease.

Findings from this review could contribute to primary prevention for those in danger of calculus disease [30,31,32,55,56].

## 6. Discussion and Conclusions

**Figure 1:**
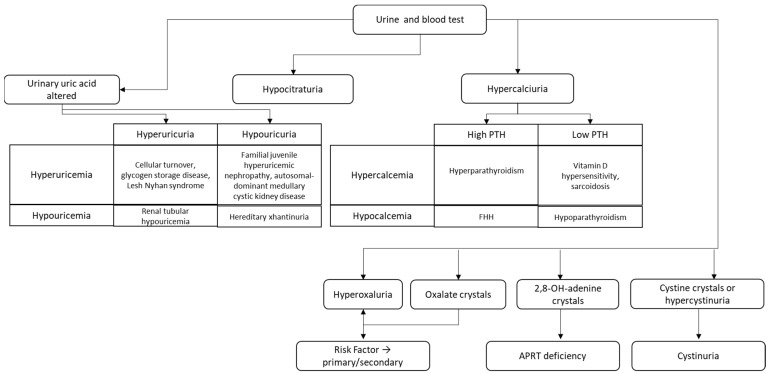
Algorithm to diagnose and evaluate pediatric urolithiasis, adapted from Marra et al. [46].

UL caused by dehydration may have a possible economic and sociological impact on cognitive and physical performance. Mild dehydration could be associated with less efficient knowledge acquisition, especially during infancy and childhood, and hence diminished performance in school [57]. Because it seems to be a risk factor for extremely frequent pathological disorders, including nephrolithiasis, which affects around 5 to 10% of the global population and has large clinical and financial implications, dehydration may even have a significant impact on public health. These factors suggest that conducting epidemiological and extensive clinical studies aimed at improving medical practice and supervision in pediatric patients will be of great interest. Assessing hydration status in a large and, therefore, representative sample seems to be of great relevance for the diagnosis and therapy of UL patients. Identifying risk factors as well as effective therapies, including an appropriate hydration, could have important implications for diagnostic and therapeutic planning in healthcare facilities (See Figure 1) [58]. 

While assessing children with UL, it must be considered that differences in metabolic causes and recurrence have been observed between infants and children, and, hence, treatment must be guided not only by the underlying causes but also by the age of the patient and his or her nutrition [59].

So far, no ideal and consensual or specific method has been established to evaluate the hydration status of children. Due to the requirement of a baseline number, weight change appears to be difficult to measure in this situation because plasma osmolality directly reflects intracellular osmolality and constitutes a reliable marker to assess acute hydration changes but cannot represent chronic hydration status as it changes constantly. Among the urinary markers, urine color is probably the least sensitive marker. Urine osmolality and particularly urine denseness may be used to assess hydration status. Although 24 h urine collection is currently the gold standard to assess urine concentration, it is a challenging procedure that is difficult to use in large-sample studies. First morning urine or afternoon urinary spot samples could even be used, with the former being easier to standardize and the latter being more representative of the whole-day water balance. Knowledge about the daily repartition of fluid intake is required to research urinary markers. Understanding the advantages and limitations of using each hydration status marker could be a key point to conduct large-sample studies on hydration status.

## 7. Recommendations

As a conclusion from the findings of previous studies and our literature review, dietary intake plays a vital role in preventing kidney stone formation in juveniles. Moreover, children who are at risk of UL may benefit from a tailored dietary therapy based on individual nutritional needs [30].

Children with hereditary risk factors for UL are considered high-risk patients and should be monitored closely. More genetic studies are required to identify such hereditary risk factors and allow for their timely screening. In third-world countries, where medical and dietary knowledge and education are often scarce and the availability of clean drinking water is limited, educational awareness programs may contribute to a reduction in the prevalence of UL.

Although knowledge of the pathophysiology of UL has been expanded in recent years, studies of UL in the pediatric population are still scarce and, hence, the most suitable treatment remains elusive. Future studies must focus on effective and accessible treatment options for children with UL, and practice guidelines should be developed for pediatric nephrologists and urologists.

## Figures and Tables

**Table 1:** Dietary recommendations for patients with calcium oxalate stones [34,35,36,37].

Urinary Risk Factor	Recommendation
Urine volume (<2.0 L/24 h)	Fluid intake that achieves urine volume of 2.0 to 2.5 L/24 h Alkalizing beverages
Hypercalciuria (>0.1 mmol/kg body weight/24 h)	Calcium intake:1000 to 1200 mg/day Protein intake: 0.8 to 1.0 g/kg normal body weight/day Sodium chloride intake: <6 g/day Increased intake of vegetables and fruit
Hyperoxaluria (>0.5 mmol/24 h)	Low dietary oxalate intake Calcium intake: 1000 to 1200 mg/day (IH) Calcium supplementation (EH)
Hyperuricosuria (>4 mmol/24 h)	Protein intake: 0.8 to 1.0 g/kg normal body weight/day Reduced dietary purine intake Increased intake of vegetables and fruit
Hypocitraturia (<1.7 mmol/24 h)	Protein intake: 0.8 to 1.0 g/kg normal body weight/day Increased intake of vegetables and fruit

## Data Availability

Not applicable.
